# Dr. George S. Palmer

**Published:** 1900-05

**Authors:** 


DR. GEORGE S. PALMER, of Buffalo, U. of B. '86, died at his home, 325 Franklin Street, in this city, April 14, 1900, aged 38 years. Dr. Palmer had been in poor health for some months, the immediate cause thereof being a fall he sustained last autumn. He had been engaged in the practice of medicine in Buffalo since his graduation. He was unmarried and is survived by his mother, his only near kin.

